# Identifying vital nodes in complex networks by adjacency information entropy

**DOI:** 10.1038/s41598-020-59616-w

**Published:** 2020-02-14

**Authors:** Xiang Xu, Cheng Zhu, Qingyong Wang, Xianqiang Zhu, Yun Zhou

**Affiliations:** Science and Technology on Information Systems Engineering Laboratory, National University of Defense Technology, Changsha, 410072 China

**Keywords:** Information technology, Software

## Abstract

Identifying the vital nodes in networks is of great significance for understanding the function of nodes and the nature of networks. Many centrality indices, such as betweenness centrality (BC), eccentricity centrality (EC), closeness centricity (CC), structural holes (SH), degree centrality (DC), PageRank (PR) and eigenvector centrality (VC), have been proposed to identify the influential nodes of networks. However, some of these indices have limited application scopes. EC and CC are generally only applicable to undirected networks, while PR and VC are generally used for directed networks. To design a more applicable centrality measure, two vital node identification algorithms based on node adjacency information entropy are proposed in this paper. To validate the effectiveness and applicability of the proposed algorithms, contrast experiments are conducted with the BC, EC, CC, SH, DC, PR and VC indices in different kinds of networks. The results show that the index in this paper has a high correlation with the local metric DC, and it also has a certain correlation with the PR and VC indices for directed networks. In addition, the experimental results indicate that our algorithms can effectively identify the vital nodes in different networks.

## Introduction

The vital nodes in networks are the nodes that have great impacts on the network structure and function^1^. Previous studies have described many centralities that can rank the nodes in networks, such as degree centrality^2^, eccentricity^3^, closeness centricity^4^, betweenness centrality^5–7^, eigenvector centrality^8^ and PageRank^9^. Identifying the influential nodes in networks is not only of theoretical significance but also of practical value. For example, identifying the important junctions in traffic networks can prevent the paralysis of traffic networks caused by traffic congestion. Locking key sources in virus transmission networks can significantly reduce the speed and scope of virus transmission. These examples and others are all related to identifying the vital nodes in networks. The paper of Gino *et al*. applied the optimal percolation theory to predict the influential nodes in memory networks^10^.

Considering that the local metrics have lower computational complexity and the global metrics have higher computational accuracy, in recent work, many vital node identification methods that consider both local and global metrics have been proposed. A semi-local metric that balances the accuracy and efficiency was proposed by Chen *et al*.^11^. Another neighbourhood centrality that takes into account the importance of a node and its neighbours’ was proposed^12^. In the paper by Yu *et al*.^13^, an improved method called improved structural holes (ISH) that identifies the key nodes in complex networks was proposed; unlike the eccentricity and betweenness centrality, this method can be applied to large-scale and disconnected networks. Zhang *et al*.^14^ presented an effective method named VoteRank to identify a set of dispersive spreaders with the best spreading ability. By considering the propagation probability, Ma *et al*.^15^ proposed a new algorithm named hybrid degree centrality (HC) to improve the local metrics and combined it with degree centrality. Lü *et al*.^16^ gave a complete overview of the vital node identification methods in recent years.

In addition to the above perspectives, many other related references use network dynamics to study the importance of nodes in networks. Lü *et al*.^17^ devised an adaptive and parameter-free algorithm, the LeaderRank, to measure the influence of users in social networks, and the experimental results show that the algorithm is more efficient than PageRank and more robust to noisy data. Min^18^ proposed a method using a message-passing approach for identifying the most influential spreaders in networks and found that the method can be easily applied to unweighted and weighted networks. Liu *et al*.^19^ presented dynamics-sensitive (DS) centrality for locating influential nodes by combining the topological and dynamic characteristics of the networks. Zhang *et al*.^20^ designed a multiscale node-importance method to measure the importance of nodes in the process of network dynamics according to different network scales. Many related studies only identify the vital nodes for a certain type of network, such as refs. ^21–23^ that only study node identification for weighted networks, Chen *et al*.^24^ who proposed an identification method for directed networks, reference^25^ that mined the vital nodes in directed weighted complex networks, and Edgar *et al*.^26^ who used Kuramoto and Ising dynamics to study the central role of peripheral nodes in directed networks. The last paper argued that a large key component does not uniquely ensure the emergence of collective phenomena, as it does for undirected networks.

To identify the vital nodes for different types (unweighted-undirected, unweighted-directed, weighted-undirected and weighted-directed) of networks, we propose an adjacency information entropy method to identify the vital nodes in different networks by considering the weights and directions of the edges in networks. For weighted networks, the node strength is used instead of the node degree. For directed networks, in order to refine the influence of the out-degree and in-degree on the node importance, we set the influence coefficient *θ* of the in-degree value. By adjusting the size of *θ*, we can control the different influences of the out-degree and in-degree on nodes.

The rest of this paper is organised as follows. In section 2, we provide detailed representations of the different types of networks. In section 3, two vital node identification algorithms and three related definitions are proposed. In section 4, four empirical experiments on the independent parts, largest components, network efficiency and correlations analysis are carried out, and the experimental results are compared and explained. Finally, the conclusion and future works are presented in section 5.

## Representations

In this paper, we study vital node identification in four different types of networks, namely, unweighted-undirected networks, unweighted-directed networks, weighted-undirected networks and weighted-directed networks. Obviously, the representations of the different networks and the calculations of the related metrics in the networks are different.

### Four different types of networks

**Figure 1:**
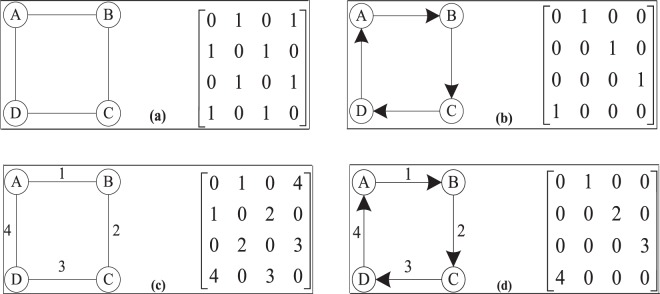
Four different types of networks and their corresponding adjacency matrices. (**a**) An unweighted-undirected network and its adjacency matrix. (**b**) An unweighted-directed network and its adjacency matrix. (**c**) A weighted-undirected network and its adjacency matrix. (**d**) A weighted-directed network and its adjacency matrix.

Usually, an unweighted network is represented by *G* = (*V*, *E*), where *V* = {*v*_1_, *v*_2_, ⋯, *v*_*n*_} and ∣*V*∣ is the number of nodes in the network. *E* = {*e*_1_, *e*_2_, ⋯ , *e*_*m*_} and ∣*E*∣ is the number of edges in the network. An adjacency matrix is used to represent the connections between the nodes in the network, and the topology of the network can be obtained by using the adjacency matrix. In Fig. 1(a), the left figure is an unweighted-undirected network and the right figure is its corresponding adjacency matrix. It is obvious that the adjacency matrix of undirected network is a symmetric matrix.

The degree of nodes in unweighted-undirected networks can be calculated by ${k}_{i}={\sum }_{j=1}^{m}{a}_{ij}$, where *j* is the neighbour of node *i* and *m* is the number of neighbours of node *i*. *a*_*ij*_ = 1 if there is an edge between node *i* and node *j*, and otherwise it is 0.

Unlike unweighted-undirected networks, the edges between the nodes in unweighted-directed networks have directions. The asymmetry of the adjacency matrix can reflect the directions of the edges in networks. We can see that the matrix in Fig. 1(b) is different from the matrix in Fig. 1(a). There are two kinds of degrees of nodes in directed networks, namely, in-degree and out-degree. In directed networks, the in-degree of a node is the number of edges from its neighbours that point to it, and the out-degree of a node is the number of edges of the node that point to its neighbours. These two kinds of degrees can be calculated by Eqs. 1 and 2, respectively.

1 $${k}_{i}^{in}=\{\begin{array}{cc}{\sum }_{j\in {\Gamma }_{i}}{a}_{ji} & \,{\rm{i}}{\rm{f}}\,{\rm{t}}{\rm{h}}{\rm{e}}\,{\rm{n}}{\rm{e}}{\rm{t}}{\rm{w}}{\rm{o}}{\rm{r}}{\rm{k}}\,{\rm{i}}{\rm{s}}\,{\rm{u}}{\rm{n}}{\rm{w}}{\rm{e}}{\rm{i}}{\rm{g}}{\rm{h}}{\rm{t}}{\rm{e}}{\rm{d}}\,\\ {\sum }_{j\in {\Gamma }_{i}}{w}_{ji} & \,{\rm{i}}{\rm{f}}\,{\rm{t}}{\rm{h}}{\rm{e}}\,{\rm{n}}{\rm{e}}{\rm{t}}{\rm{w}}{\rm{o}}{\rm{r}}{\rm{k}}\,{\rm{i}}{\rm{s}}\,{\rm{w}}{\rm{e}}{\rm{i}}{\rm{g}}{\rm{h}}{\rm{t}}{\rm{e}}{\rm{d}}\,\end{array}$$

2 $${k}_{i}^{out}=\{\begin{array}{cc}{\sum }_{j\in {\Gamma }_{i}}{a}_{ij} & \,{\rm{i}}{\rm{f}}\,{\rm{t}}{\rm{h}}{\rm{e}}\,{\rm{n}}{\rm{e}}{\rm{t}}{\rm{w}}{\rm{o}}{\rm{r}}{\rm{k}}\,{\rm{i}}{\rm{s}}\,{\rm{u}}{\rm{n}}{\rm{w}}{\rm{e}}{\rm{i}}{\rm{g}}{\rm{h}}{\rm{t}}{\rm{e}}{\rm{d}}\,\\ {\sum }_{j\in {\Gamma }_{i}}{w}_{ij} & \,{\rm{i}}{\rm{f}}\,{\rm{t}}{\rm{h}}{\rm{e}}\,{\rm{n}}{\rm{e}}{\rm{t}}{\rm{w}}{\rm{o}}{\rm{r}}{\rm{k}}\,{\rm{i}}{\rm{s}}\,{\rm{w}}{\rm{e}}{\rm{i}}{\rm{g}}{\rm{h}}{\rm{t}}{\rm{e}}{\rm{d}}\,\end{array}$$

Generally, the calculation of the degree in directed networks adds the in-degree to the out-degree. Here, we consider that the in-degree and out-degree of nodes have different effects on nodes^27^. Then, the degree of nodes in directed networks can be calculated by Eq. 3, where *θ* is the influence coefficient of the nodes’ in-degree, and in this paper, we set *θ* = 0.75.

3 $${k}_{i}^{unweighted}=\theta {k}_{i}^{in}+(1-\theta ){k}_{i}^{out}=\theta \mathop{\sum }\limits_{j=1}^{m}{a}_{ji}+(1-\theta )\mathop{\sum }\limits_{j=1}^{m}{a}_{ij}$$

A weighted network can be represented by *G* = (*V*, *E*, *W*), where *W* is the adjacency weighted matrix of the network. The weights of the connected edges in weighted networks are not only 0 or 1, and edges’ weights can reflect the strength of the relationships between nodes. Figure 1(c) presents a weighted-undirected network and the corresponding adjacency matrix. The degree of nodes in weighted-undirected networks can be obtained by ${k}_{i}={\sum }_{j=1}^{m}{w}_{ij}$, where *w*_*ij*_ is the weight of the edge between node *i* and node *j*.

**Figure 2:**
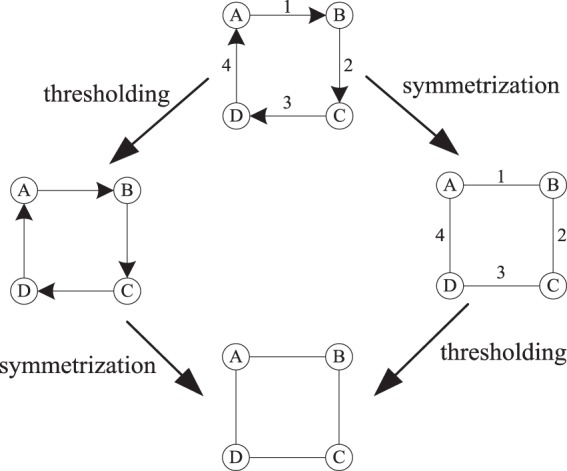
Relationships among the four different types of networks. Directed networks can obtain their corresponding undirected networks via symmetrisation, and weighted networks can obtain their corresponding unweighted networks via thresholding.

Weighted-directed networks are the most complicated of the four types of networks. Figure 1(d) shows a simple weighted-directed network and its adjacency weighted matrix. According to the above degree calculation method for weighted networks and directed networks, naturally, the degree of nodes in weighted-directed networks can be obtained by Eq. 4. Figure 2 illustrates the relationships among the four different types of networks.

4 $${k}_{i}^{weighted}=\theta {k}_{i}^{in}+(1-\theta ){k}_{i}^{out}=\theta \mathop{\sum }\limits_{j=1}^{m}{w}_{ji}+(1-\theta )\mathop{\sum }\limits_{j=1}^{m}{w}_{ij}$$

## Methods

### Related definitions

To identify the vital nodes in different types of networks, we propose three definitions as follows.

**Figure 3:**
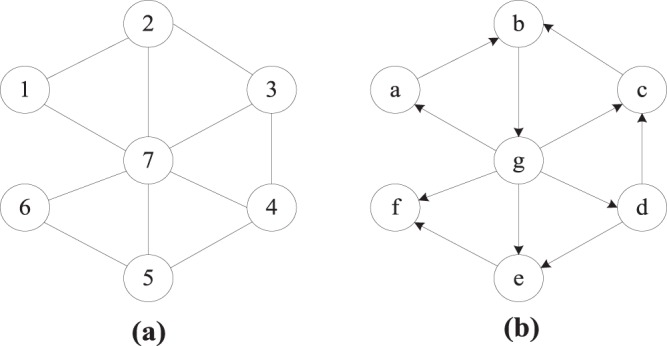
Two example networks with 7 nodes and 11 edges. **(a)** An unweighted-undirected network. **(b)** An unweighted-directed network.

**Definition 1**. Adjacency degree *A*_*i*_. We define the adjacency degree of nodes in undirected networks by considering its nearest neighbours as ${A}_{i}={\sum }_{j\in {\Gamma }_{i}}{k}_{j}$, where *j* is the neighbour of node *i*, *Γ*_*i*_ is the set of neighbours of node *i*, and *k*_*j*_ is the degree of node *j*. For example, in Fig. 3(a), *A*_1_ = *k*_2_ + *k*_7_ = 3 + 6 = 9. In directed networks, the adjacency degree of nodes is defined as follows (Eq. 5), where ${k}_{{j}_{in}}$ is the number of edges that point to node *j* from node *i*, and ${k}_{{j}_{out}}$ is the number of edges from node *j* that point to node *i*. For example, in Fig. 3(b), *A*_*b*_ = *θ*(*k*_*a*_ + *k*_*c*_) + (1 − *θ*)*k*_*g*_ = *θ* * (1 + 1.75) + (1 − *θ*) * 2 = 2.5625.

5 $${A}_{i}=\theta \sum _{j\in {\Gamma }_{i}}{k}_{{j}_{in}}+(1-\theta )\sum _{j\in {\Gamma }_{i}}{k}_{{j}_{out}}$$

**Definition 2**. Selection probability ${P}_{{i}_{j}}$. We define the selection probability of node *i* in the network by considering the probability that it will be selected by its neighbour *j*, and the calculation formula is Eq. 6.

Taking from the idea from information theory, a certain node in the network is taken as the information source point, and its neighbouring nodes are taken as the target points. In the process of information transmission or disease transmission, the information source point and infected person will select the target point among its neighbouring nodes for information transmission or disease infection. The probability that the target nodes are selected is called the selection probability. This definition considers the importance of the selected nodes, that is, the influence of the degrees of the selected node in the selection process.

6 $${P}_{{i}_{j}}={k}_{i}/{A}_{j},(j\in {\Gamma }_{i})$$

For example, in Fig. 3(a)${P}_{{1}_{2}}={k}_{1}/{A}_{2}={k}_{1}/({k}_{1}+{k}_{3}+{k}_{7})=2/(2+3+6)\approx 0.23$. Similarly, ${P}_{{1}_{7}}={k}_{1}/{A}_{7}=$ ${k}_{1}/({k}_{1}+{k}_{2}+{k}_{3}+{k}_{4}+{k}_{5}+{k}_{6})=2/(2+3+3+3+3+2)=0.125$.

**Definition 3**. Adjacency information entropy *E*_*i*_. We define the adjacency information entropy of nodes in undirected networks as Eq. 7 and that in directed networks as Eq. 8.

7 $${E}_{i}=-{\sum }_{j\in {\Gamma }_{i}}({P}_{{i}_{j}}lo{g}_{2}{P}_{{i}_{j}})$$

8 $${E}_{i}={\sum }_{j\in {\Gamma }_{i}}| (-{P}_{{i}_{j}}lo{g}_{2}{P}_{{i}_{j}})| $$

### Vital node identification algorithms

According to the characteristics of the four different types of networks, the proposed algorithms in this paper can be applied to different networks. Before the algorithms can be applied, we need to obtain the adjacency matrix ***A*** or the adjacency weighted matrix ***W***of the network. From the above definitions, we can rank the nodes in the network by the value of the node’s adjacency information entropy (*E*_*i*_), and the specific algorithms step are as follows.

**Algorithm 1:**
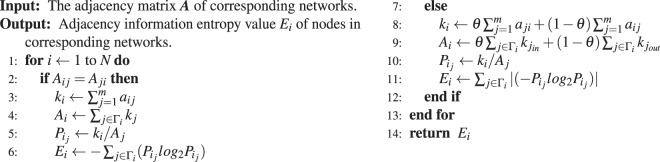
Calculate *F*_*i*_ for unweighted networks.

**Algorithm 2:**
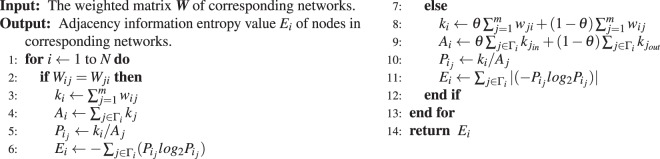
Calculate *F*_*i*_ for weighted networks.

## Results and Discussion

To verify the accuracy and applicability of our proposed algorithms, four different kinds of networks are employed, which include (1) unweighted-undirected networks (UUNs), (2) unweighted-directed networks (UDNs), (3) weighted-undirected networks (WUNs), and (4) weighted-directed networks (WDNs). The statistical properties of the studied networks are listed in Table 1. With respect to the unweighted-undirected networks, the Astro network is a collaboration network of astrophysics scientists^28^; the CA network is a large connected component of the arXiv collaboration network in high-energy physics theory^29^; the Facebook network is an anonymised social networks with 4039 users, where the data can be downloaded in http://snap.stanford.edu/data/; and the Hamster network is a friendship and family connections network among website users^30^. With respect to the unweighted-directed networks, the Email network includes 1133 email users of the University at Rovira i Virgili, URV^31^; the PGP network is a communication network^32^; Router is a topological network of the Internet^33^; and the Wiki-Vote network is a who-votes-on-whom network from Wikipedia, where the data can be downloades from http://snap.stanford.edu/data/. With respect to the weighted-directed networks, the data of the P2P and PHD networks can be obtained at http://vlado.fmf.uni-lj.si/pub/networks/data/.

**Table 1:** The statistical properties of the four kinds of complex networks, where ***n*** and ***m*** are the total numbers of nodes and edges, respectively.  < ***k*** >  and  < ***d*** >  denote the average degree and the average distance respectively, and ***C*** denotes the clustering coefficient.

UUNs	*n*	*m*	< *k* >	< *d* >	*C*
Astro	14845	239304	16.12	4.798	0.715
CA	8638	49612	5.743	5.945	0.580
Facebook	4039	88234	43.69	3.693	0.617
Hamster	2000	32194	16.09	3.589	0.573
**UDNs**	***n***	***m***	< ***k*** >	< ***d*** >	***C***
Email	1133	5451	4.811	3.715	0.110
PGP	10680	24340	2.279	4.050	0.133
Router	5022	6258	1.246	3.973	0.006
Wiki-Vote	7115	103689	14.57	3.341	0.081
**WUNs**	***n***	***m***	< ***k*** >	< ***d*** >	***C***
Astro	14845	239304	1256.6	4.798	0.715
CA	8638	49612	448.78	5.945	0.580
Facebook	4039	88234	1113.7	3.693	0.617
Hamster	2000	32194	1257.2	3.589	0.573
**WDNs**	***n***	***m***	< ***k*** >	< ***d*** >	***C***
Email	1133	5451	4.811	3.715	0.110
P2P	6301	20777	29.663	6.632	0.005
PHD	1025	1043	8.956	3.429	0.002
Router	5022	6258	1.246	3.973	0.006

**Figure 4:**
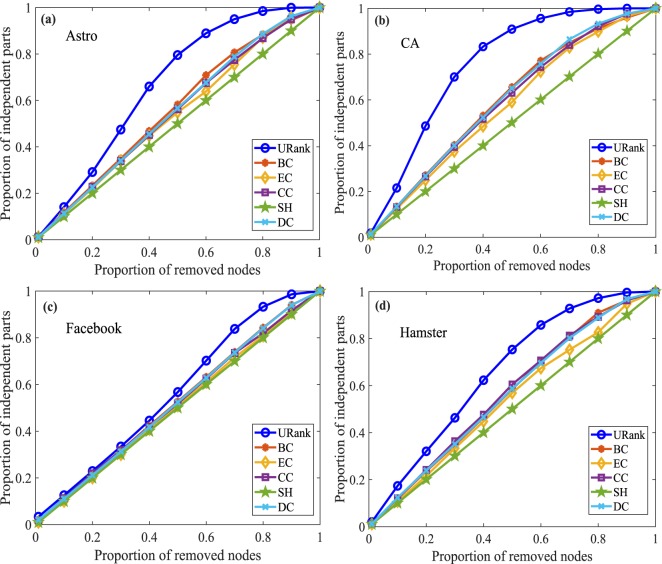
Independent parts experiments for unweighted-undirected networks. In (**a**), when the proportion of removed nodes reaches approximately 10%, the performance of URank is better than the other metrics; and when more than 20% of the nodes are removed, URank performs better and better until approximately 60% of the nodes are removed. Panel (**b**) clearly shows the performance of URank compared with the other metrics. In (**c**), it can be seen that there is little difference among the different metrics when removing the first 40% of the nodes. Panel (**d**) shows that the performance of URank is more uniform.

**Figure 5:**
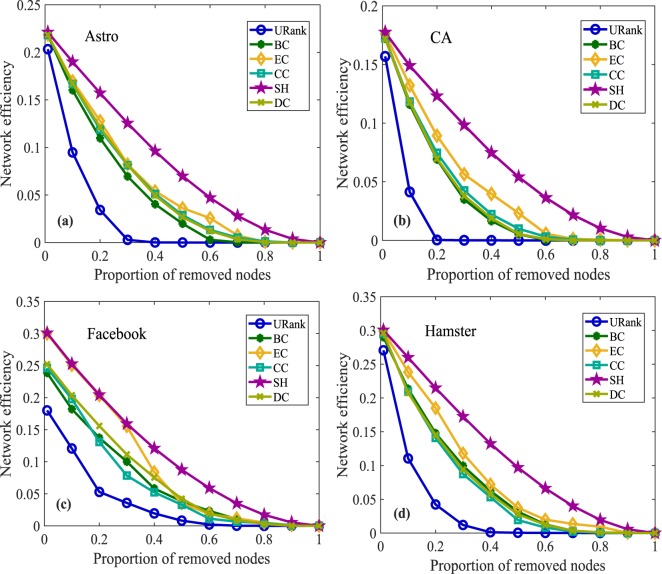
Network efficiency experiments for undirected networks. In (**a**), as the proportion of removed nodes increases, the network efficiency gradually declines after removing the nodes ranked by different metrics. Node removal ranked by URank makes the network efficiency decrease the fastest, and the network efficiency corresponding to URank is the smallest when the same proportion of nodes are removed. In (**b**), after removing approximately 20% of the nodes ranked by the algorithm in this paper, the network efficiency is reduced to the lowest, while the other metrics need to remove approximately 70% of the nodes. Panel (**c**) shows that the performance of our metric is more uniform. Panel (**d**) clearly shows the performance of URank compared with the other metrics.

We will verify the accuracy of our algorithms by computing the proportion of independent parts of networks by removing the different proportions of nodes. Obviously, the larger the proportion of independent parts is, the more seriously the network is destroyed, and the higher the identification accuracy of vital nodes is. For undirected networks, we selected five other centralities as benchmark indices, namely, betweenness centrality (BC), eccentricity centrality (EC), closeness centrality (CC), structural holes centrality^34^ (SH) and degree centrality (DC). For simplicity, we call our algorithm for unweighted-undirected networks URank and that for weighted-undirected networks WRank. The X-axis is the different proportions of removed nodes. The Y-axis is the proportions of independent parts of corresponding networks. The results of the different centralities after removing different proportions of ranked nodes in the four different unweighted-undirected networks are shown in Fig. 4. From Figure 4, it is clear that our algorithm is more significant. Similarly, Supplementary Fig. S1 shows the experimental results for weighted-undirected networks. In addition, according to the importance of the nodes, we also remove the different proportions of nodes from high to low to test the efficiency of the undirected networks. As is well known, the higher the network efficiency is, the smaller the average distance between the nodes in the network is. If the removed nodes cause the network efficiency to decline more, the impact of the removed nodes on the network is greater, the removed nodes are more important. Figure 5 shows the experimental results of the network efficiency curves of undirected networks after removing different proportions of nodes.

For directed networks, we also selected five other centralities as benchmark indices, namely, PageRank centrality (PR), eigenvector centrality (VC), eccentricity centrality (EC), closeness centrality (CC) and degree centrality (DC). Similarly, for simplicity, we call our algorithm for unweighted-directed networks DRank and that for weighted-directed networks WDRank. Supplementary Figs. S2 and S3 show the independent parts experimental results in unweighted-directed networks and weighted-directed networks, respectively.

**Figure 6:**
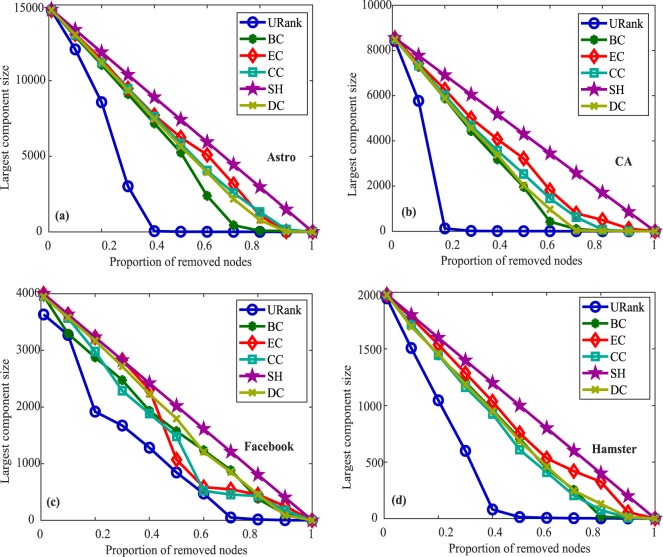
Largest component experiments for unweighted-undirected networks. In (**a**), after removing approximately 40% of the nodes ranked by our algorithm, the largest component size is reduced to the lowest, while the other metrics need to remove approximately 80% or 90% of the nodes. Panel (b)clearly shows that the performance of URank compared with other metrics. In (**c**), the figure shows the performance of our metric is more uniform, and there is little difference in the removal ratio of nodes when each metric makes the largest component size reach the lowest. In panel (d), when removing the first 40% of the nodes, our metric makes the largest component size decline the fastest until it reaches the minimum.

To further prove the effectiveness and applicability of the proposed algorithms, we implemented the largest component experiments using the four different types of networks. When some nodes in the network are deleted according to the importance of the nodes, different sized components will be formed. If the size of the component is smaller, the removed nodes are more destructive to the original network. The largest component experiments can illustrate the accuracy of the vital node identification algorithms from another perspective. The X-axis is the different proportions of removed nodes. The Y-axis is the largest component sizes of the different networks when the corresponding proportion nodes were removed. Figure 6 shows the experimental results for unweighted-undirected networks. Figure 6, shows that the URank algorithm performs well for most networks. The results of the same largest component experiments for unweighted-directed networks, weighted-undirected networks and weighted-directed networks are shown in Supplementary Figs. S5–S6, respectively.

**Figure 7:**
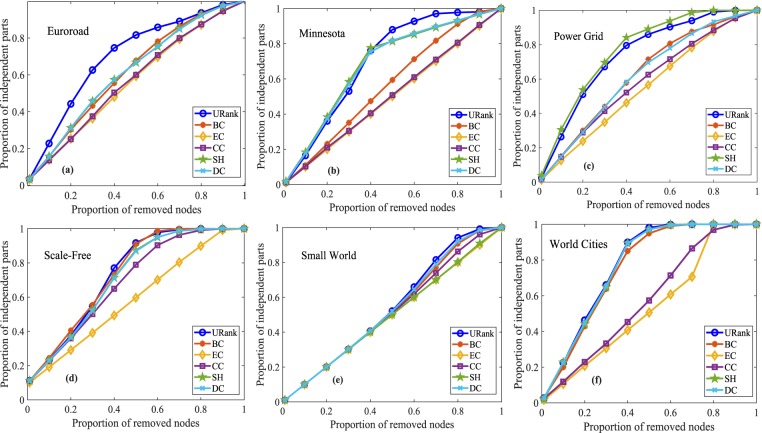
Independent parts experiments for spatial networks and classical networks. In (**a**), as the number of removed nodes increase, the proportion of parts corresponding to URank is significantly more than those of the other metrics. From (**b**,**d**,**e**), we can see that the performance of URank is less obvious than that in (**a**). In (**c**), URank performs better than the other metrics except the SH metric. In (**f**), the URank performs as well as DC, and it is better than the other metrics.

**Figure 8:**
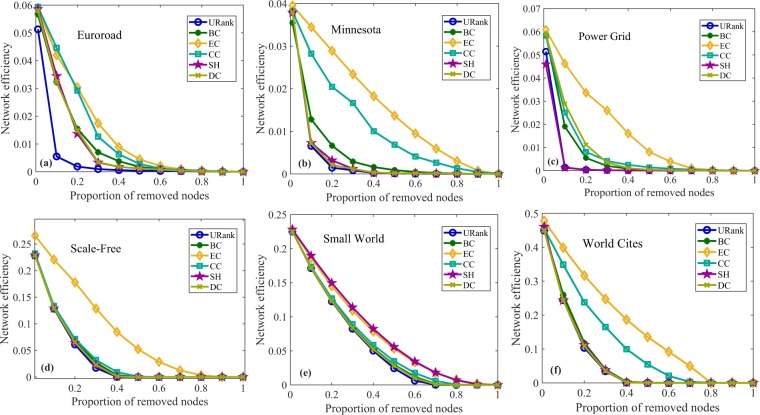
Network efficiency experiments for spatial networks and classical networks. In (**a**), compared with the other metrics, removing nodes ranked by URank makes the network efficiency decline the fastest, and the extent of the decline is also the largest. However, in (**b**–**f**), the advantage of URank is not obvious and, generally speaking, the performance of the metric in this paper is better than those of other metrics.

**Figure 9:**
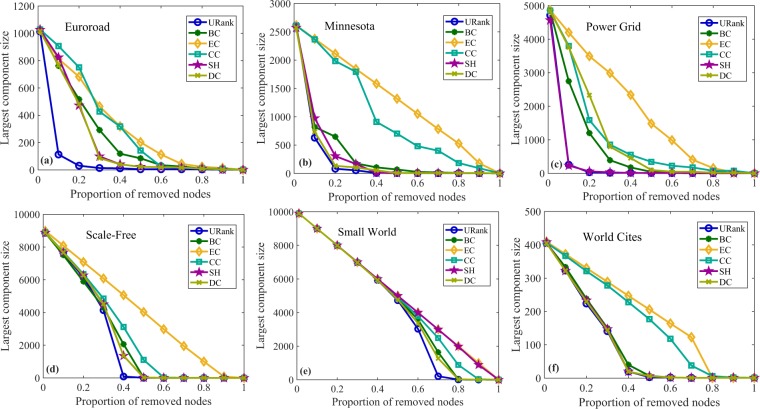
Largest component experiments for spatial networks and classical networks. In (**a**), we can see that when the nodes in the network are removed according to URank, removing approximately 10% of the nodes reduced the largest component size of the network by approximately 90%. If the nodes are removed after being ranked by other metrics, more than 30% of the nodes need to be removed to achieve the same effect. In (**b**), the performance of our metric is slightly better than other metrics. In (**c**,**f**), URank and SH perform equally well. In (**d**,**e**), the performance of URank gradually gets better as the node removal ratio increases until the largest component size of the network reaches the minimum.

To verify the applicability of our algorithms to other kinds of networks, we further carry out the three verification experiments described above using spatial networks and classical networks, such as a small world network and scale-free network, respectively. Figures 7, 8 and 9 present the results of the independent parts experiments, the network efficiency experiments and the largest component experiments, respectively. The corresponding statistical properties of the spatial networks and classical networks are listed in Table 2. Euroroad and Minnesota are road networks, and the data can be downloaded from http://networkrepository.com/road.php. Power Grid^35^ contains an undirected unweighted representation of the topology of the Western States Power Grid of the United States, which was compiled by Duncan Watts and Steven Strogatz. The data are downloaded from the web site of Prof. Duncan Watts at Columbia University, http://cdg.columbia.edu/cdg/datasets. The Scale-Free and Small World networks are generated by the Pajek software. World Cites is a network of 415 cities, and the data can be obtained from http://www-personal.umich.edu/mejn/netdata/.

**Table 2:** The statistical properties of spatial networks and classical networks, where ***n*** and ***m*** are the total numbers of nodes and edges, respectively.  < ***k*** >  and  < ***d*** >  denote the average degree and the average distance, respectively, and ***C*** denotes the clustering coefficient.

Other networks	*n*	*m*	< *k* >	< *d* >	*C*
Euroroad	1174	1417	2.414	18.371	0.020
Minnesota	2642	3303	2.500	35.349	0.017
Power Grid	4941	6594	2.669	18.989	0.107
Scale-Free	10000	187396	18.740	3.223	0.042
Small World	10000	100000	10.000	4.443	0.090
World Cites	415	7518	36.231	2.238	0.003

To investigate the relations between our algorithms and other centralities in different networks, we conducted correlation analysis experiments. We use the Kendall’s Tau to describe the relationship between different centralities. The relevant definitions are as follows^36^.

Assuming that two random variables are *X* and *Y* (they can also be regarded as two sets), their number of elements is *N*, where *X*_*i*_ and *Y*_*i*_ represent the *i*-th element of each random variable, respectively. The corresponding elements in *X* and *Y* form an element pair set *XY*, which contains the elements (*X*_*i*_, *Y*_*i*_)(1≤*i*≤*N*). When *X*_*i*_ > *X*_*j*_ and *Y*_*i*_ > *Y*_*j*_ or *X*_*i*_ < *X*_*j*_ and *Y*_*i*_ < *Y*_*j*_, these two elements are considered to be concordant. When *X*_*i*_ > *X*_*j*_ and *Y*_*i*_ < *Y*_*j*_ or *X*_*i*_ < *X*_*j*_ and *Y*_*i*_ > *Y*_*j*_, these two elements are considered to be discordant. When *X*_*i*_ = *X*_*j*_ or *Y*_*i*_ = *Y*_*j*_, the two elements are neither concordant nor discordant. Kendall’s Tau is defined as.

9 $$\tau =\frac{{N}_{c}-{N}_{d}}{N(N-1)/2}$$

where *N*_*c*_ and *N*_*d*_ are the number of concordant and discordant pairs, respectively. *N* is the number of nodes in the network.

**Figure 10:**
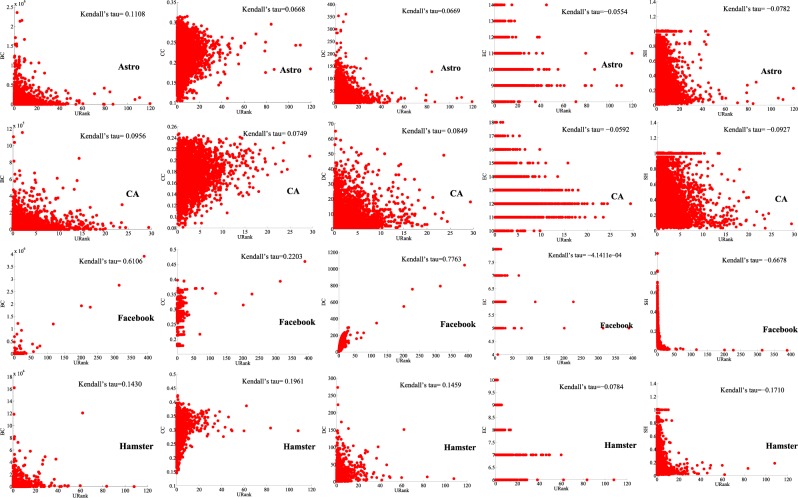
Correlation analysis experiments in unweighted-undirected networks.

**Figure 11:**
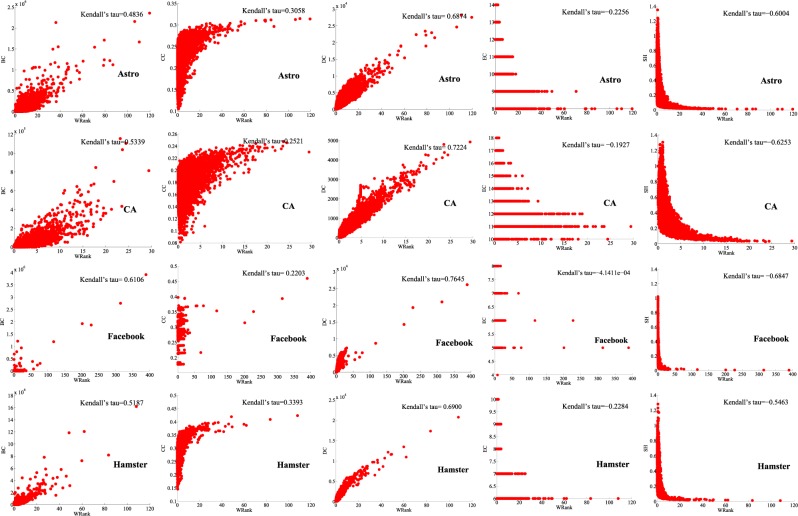
Correlation analysis experiments in weighted-undirected networks.

In undirected networks, from Figs. 10 and 11, we can see that our centrality index is negatively correlated with EC and SH, because EC considers the node with the largest distance from the node, while SH considers the constraint coefficient of the node. The smaller the constraint coefficient is, the more important the node is, contrary to our centrality index in this paper. In unweighted-undirected networks, we can see from Fig. 10 that there is no obvious correlation between our centrality index and other centralities, but in the Facebook network, our centrality index has a high positive correlation with BC, CC and DC. The reason may be that Facebook is a social network, and the propagation between nodes is similar to that of the adjacency entropy algorithms in this paper. In weighted-undirected networks (Fig. 11) and directed networks (Supplementary Figs. S7 and S8), we can clearly observe a high correlation between our centrality index and DC. The reason may be that our centrality index and DC are designed based on the local properties of nodes. Similarly, we can find that our centrality index has low correlation with BC, CC and EC in the four different types of networks because BC, CC and EC are global metrics. By comparing the correlations between our centrality index and PR, VC and other centralities in directed networks (Supplementary Figs. S7 and S8), we can find that the correlations between our centrality index and PR and VC are greater than those of other centralities (except DC) because PR and VC are both centralities designed for directed networks, while other centralities are applicable to both directed networks and undirected networks.

### Computational efficiency

The adjacency information entropy algorithm has two steps: the calculation of the adjacency degree and the adjacency information entropy. Since every node’s adjacency degree and adjacency information entropy in the network needs to be calculated, the computational complexity of the first cycle is *O*(*N*), where *N* is the number of the network nodes. In the calculation of the node adjacency degree, it is also necessary to traverse the neighbouring nodes of the network nodes; thus, the total computational complexity of our algorithm is *O*(*N*^2^). Since the metric used in this paper involves the first-order neighbour of the node, the algorithmic complexity is lower than those of the global metrics, such as betweenness centrality(BC) with complexity *O*(*MN*^3^) and closeness centrality(CC) with complexity *O*(*MN*^2^), where *M* is the number of edges in the network. The number of network nodes applied by our algorithm could be further scaled up under the High Performance Computing (HPC) environment.

## Conclusion

In this paper, we design two vital node identification algorithms for four different types of networks. By calculating and comparing the adjacency information entropy of nodes, the importance of nodes is ranked. The larger the entropy value is, the more vital the nodes are. The algorithms highlight the different characteristics of the different types of networks. For weighted networks, the strength of the nodes is used to calculate the adjacency information entropy instead of the degree of the nodes. For directed networks, the influence coefficient of a node’s in-degree and out-degree value is used, which further refines the influence of a node’s in-degree and out-degree on the node’s importance. The experimental results show that our proposed algorithms outperform several benchmark methods. In the future, we will consider identifying vital nodes for more realistic network types, including temporal networks, etc.

## Supplementary information

Supplementary Information.
