# Supplementary material for: Identifying vital nodes in complex networks by adjacency information entropy
Source: Sci Rep. 2020 Feb 14;10:2691. doi: 10.1038/s41598-020-59616-w (PMC7021909; doi:10.1038/s41598-020-59616-w)
Supplement: Supplementary file 1 — Supplementary Information. [file 41598_2020_59616_MOESM1_ESM.pdf]

Supplementary information for identifying vital nodes in complex networks by adjacency information entropy

Xiang Xu[1*], Cheng Zhu[1*], Qingyong Wang[1], Xianqiang Zhu[1], and Yun Zhou[1]

[1]Science and Technology on Information Systems Engineering Laboratory, National University of Defense Technology, Changsha, 410072, China
[*]xuxiang19@nudt.edu.cn
[*]zhucheng@nudt.edu.cn

ABSTRACT

Identifying the vital nodes in networks is of great significance for understanding the function of nodes and the nature of networks. Many centrality indices, such as betweenness centrality (BC), eccentricity centrality (EC), closeness centricity (CC), structural holes (SH), degree centrality (DC), PageRank (PR) and eigenvector centrality (VC), have been proposed to identify the influential nodes of networks. However, some of these indices have limited application scopes. EC and CC are generally only applicable to undirected networks, while PR and VC are generally used for directed networks. To design a more applicable centrality measure, two vital node identification algorithms based on node adjacency information entropy are proposed in this paper. To validate the effectiveness and applicability of the proposed algorithms, contrast experiments are conducted with the BC, EC, CC, SH, DC, PR and VC indices in different kinds of networks. The results show that the index in this paper has a high correlation with the local metric DC, and it also has a certain correlation with the PR and VC indices for directed networks. In addition, the experimental results indicate that our algorithms can effectively identify the vital nodes in different networks.

Supplementary information

Independent parts experiments in different networks

Supplementary Fig. S1 shows the experimental results in weighted-undirected networks

Supplementary Figs S2 and S3 show the independent parts experimental results in unweighted-directed networks and weighted-directed networks, respectively.

Largest component experiments in different networks

The largest component experimental results in unweighted-directed networks, unweighted-directed networks and weighted-directed networks are shown in Supplementary Figs S4, S5 and S6, respectively.

Correlation analysis experiments in different networks

Supplementary Figs S7 and S8 show the correlation analysis experimental results in unweighted-directed networks and weighted-directed networks, respectively.

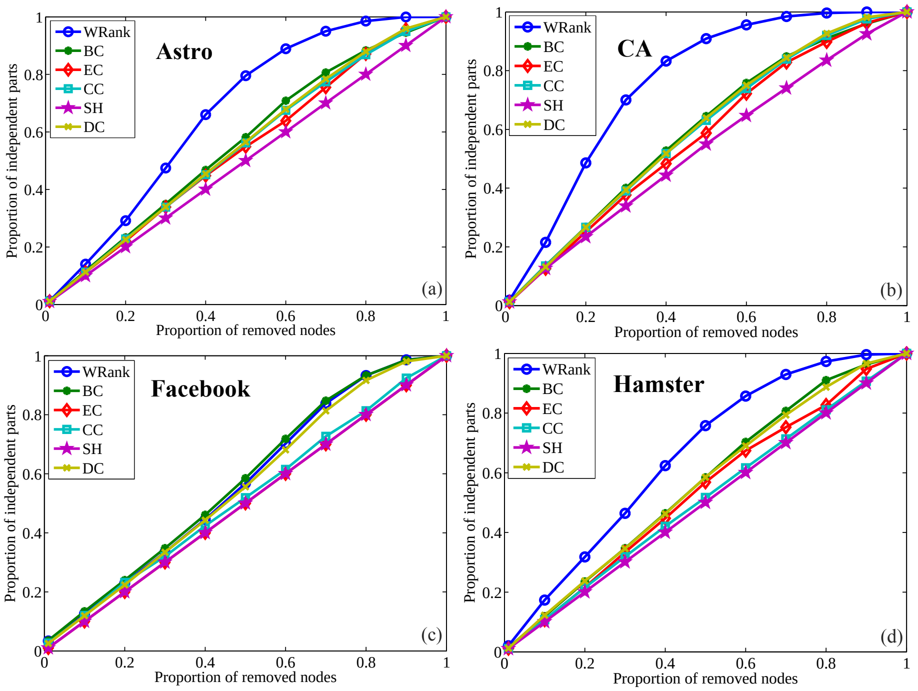

Figure S1. Independent parts experiments in weighted-undirected networks.

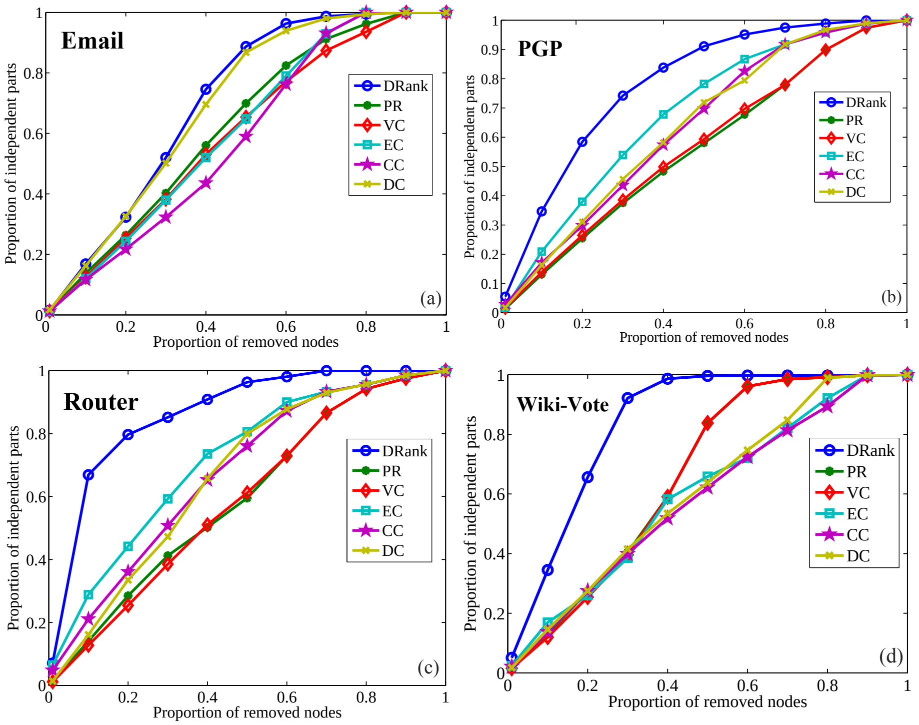

Figure S2. Independent parts experiments in unweighted-directed networks.

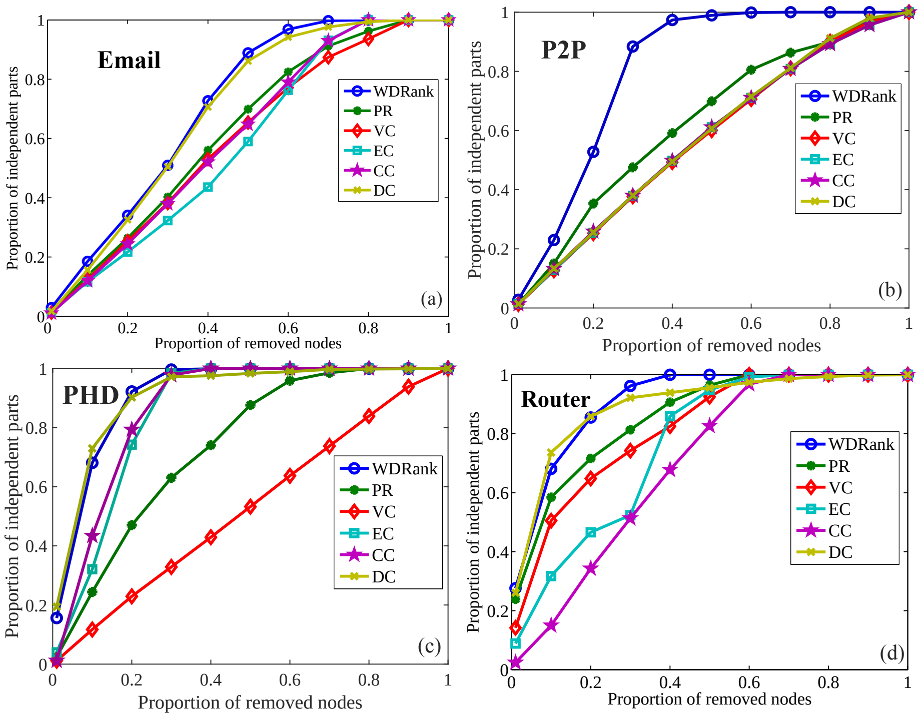

Figure S3. Independent parts experiments in weighted-directed networks.

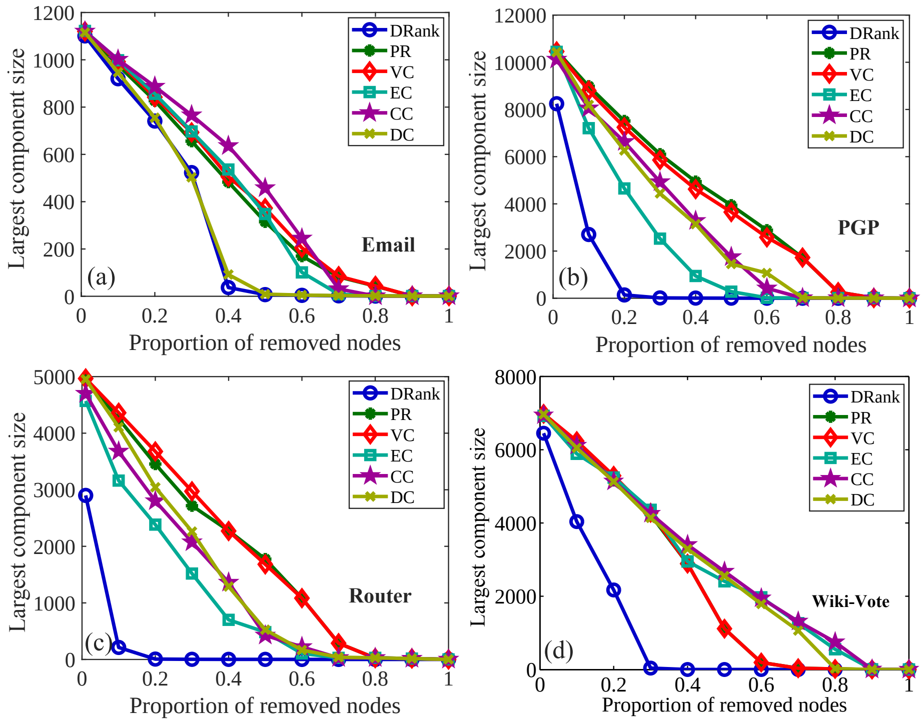

Figure S4. Largest component experiments in unweighted-directed networks.

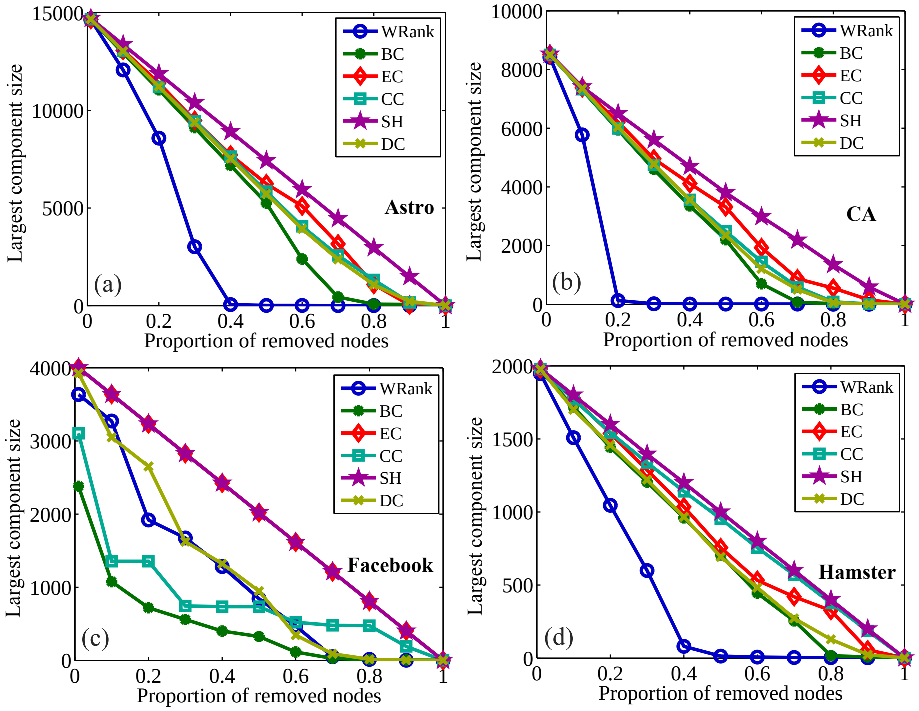

Figure S5. Largest component experiments in weighted-undirected networks.

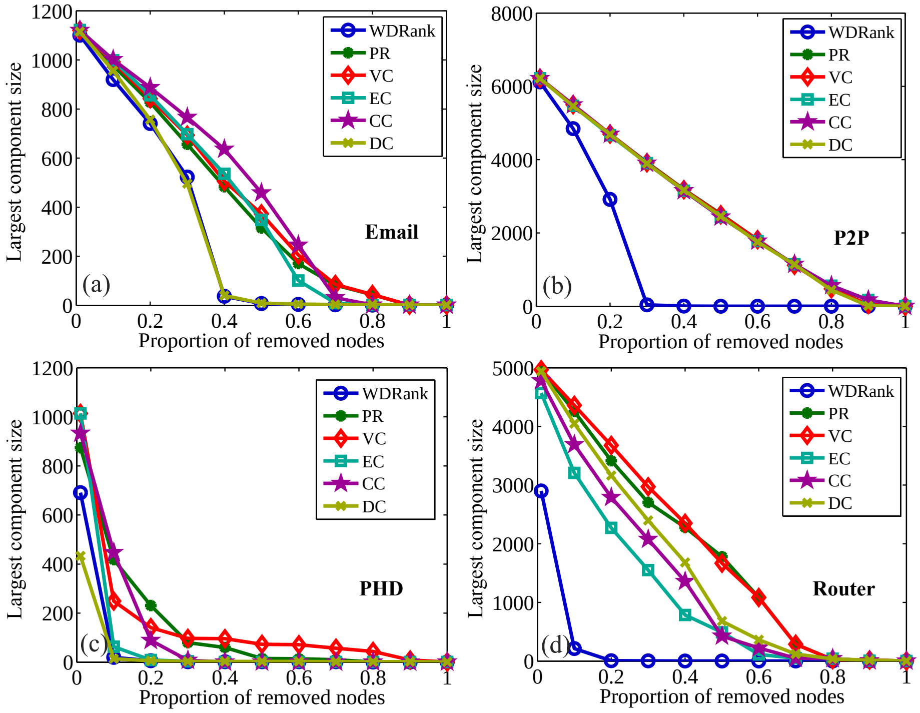

Figure S6. Largest component experiments in weighted-directed networks.

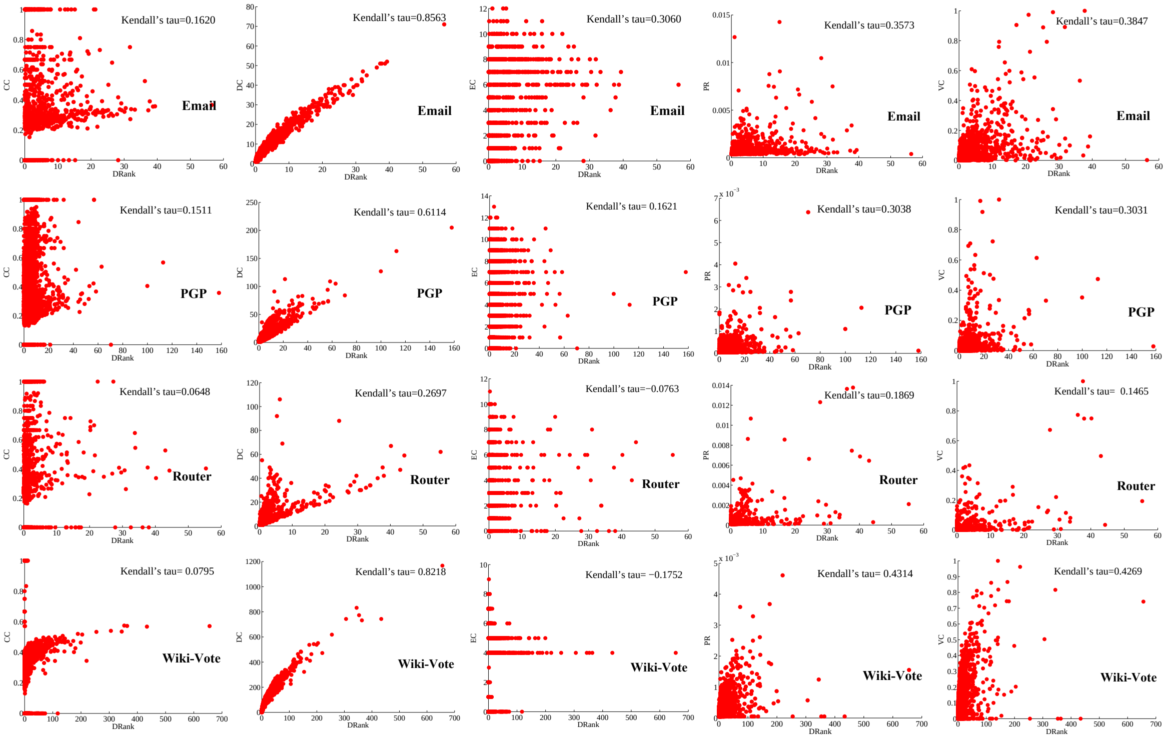

Figure S7. Correlation analysis experiments in unweighted-directed networks.

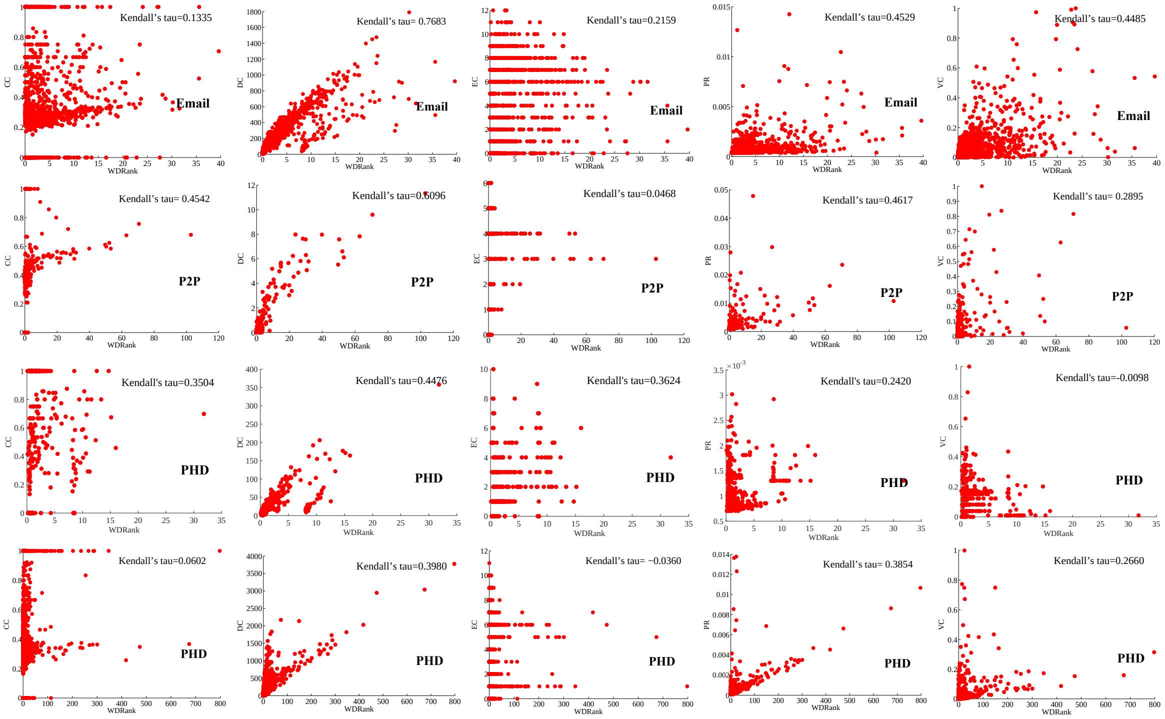

Figure S8. Correlation analysis experiments in weighted-directed networks.
